# Trends in Sigma-1 Receptor Research: A 25-Year Bibliometric Analysis

**DOI:** 10.3389/fphar.2019.00564

**Published:** 2019-05-24

**Authors:** Luz Romero, Enrique Portillo-Salido

**Affiliations:** Drug Discovery and Preclinical Development, Esteve Pharmaceuticals, Parc Científic de Barcelona, Barcelona, Spain

**Keywords:** sigma-1 receptor, bibliometrics, scopus, VOSviewer, co-occurrence analysis, co-citation analysis, co-authorship analysis

## Abstract

**Purpose:** There are previous reviews focused on Sigma-1 receptor but no bibliometric studies examining this field as a whole. This article aims to present a global view of Sigma-1 receptor research and its intellectual structure.

**Methods:** We used bibliometric indicators of a basic nature as well as techniques for the visualization and analysis of networks of scientific information extracted from Scopus database.

**Results:** In total, 1,102 articles from 1992 to 2017 were identified. The growth in the production of articles is not constant over time, with periods of stagnation of approximately 5 years. Only 247 authors have five or more publications. The authors appear grouped in relatively independent clusters, thus suggesting a low level of collaborations between those dedicated to the Sigma-1 receptor. The United States was the country with the highest production followed by Japan and Germany. Spain, Japan, and Italy showed the highest per million inhabitants ratio. The highest citation/article ratio was reached in France, United States, and Canada. The leading institutions were the University of Münster, the National Institutes of Health, ESTEVE, and INSERM. The top authors in number of publications were Wünsch-B, Schepmann-D, and Maurice-T. Hayashi-T, Su-TP and Bowen-WD showed the highest citations per article. The article by Hayashi-T and Su-TP in Cell (2007) describing the Sigma-1 receptor as a chaperone protein is the top cited reference. Cluster labeling from author co-citation analysis shows that research has been focused on specific diseases such as addiction, neuroprotection and neurodegenerative diseases, psychiatric disorders, and pain. High-frequency terms in author keywords suggest that the research efforts in some areas such as neuroimaging, cocaine addiction or psychiatric disorders have declined over time, while others such as neurodegenerative diseases or pain are currently most popular.

**Perspective:** A greater involvement of the scientific community, with an increase in the scientific production related to Sigma-1, is desirable. Additional boost needed to improve research performance is likely to come from combining data from different laboratories to overcome the limitations of individual approaches. The resulting maps are a useful and attractive tool for the Sigma-1 receptor research community, as they reveal the main lines of exploration at a glance.

## Introduction

**Figure 1:**
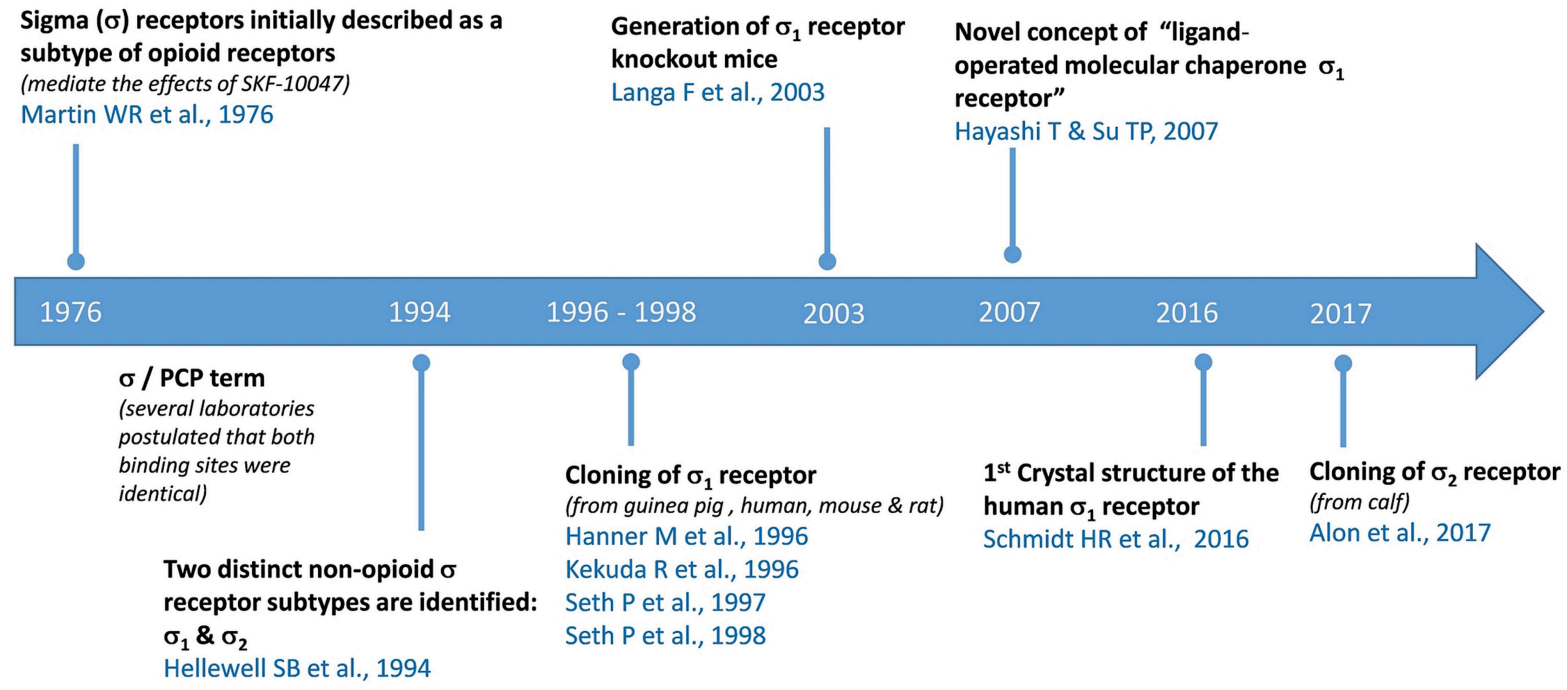
Some milestones in Sigma-1 receptor research.

The Sigma-1 receptor is considered a unique ligand-operated chaperone protein which regulates protein folding/degradation, ER/oxidative stress, and cell survival (Hayashi, 2015). Sigma-1 receptor ligands have long been expected to serve as drugs for the treatment of human diseases such as neurodegenerative disorders, depression, chronic pain, drug abuse, retinal disease, and cancer (Cobos et al., 2008; Katz et al., 2017; Kim and Maher, 2017; Maurice and Goguadze, 2017a,b; Merlos et al., 2017a,b; Sabino et al., 2017; Sanchez-Fernandez et al., 2017; Smith et al., 2018). Two subtypes of Sigma receptors have been identified, Sigma-1 and Sigma-2 (Hellewell et al., 1994). Confused with opioid receptors for many years due to the cross-reactivity of some ligands (Martin et al., 1976; Tam, 1983), the Sigma-1 receptor (also known as Sigma1, Sig1R, σ1 receptor, and several other names) was first cloned in 1996 from guinea pig liver (Hanner et al., 1996), and later from mouse kidney, human cell lines, rat brain, and mouse brain (Kekuda et al., 1996; Seth et al., 1997, 1998). The Sigma-2 receptor (Sigma2, Sig2R, σ2 receptor) was cloned very recently from calf liver (Alon et al., 2017) and identified as transmembrane protein 97 (TMEM97). Very recently, the first crystal structure of the full-length human Sig-1R was reported in a complex with two different ligands, PD144418 and 4-IBP (Schmidt et al., 2016). As shows in the Figure 1, representing a chronological view of these important milestones, the research on Sigma-1 receptor has evolved since the cloning of the receptor. The Sigma-1 receptor shares no homology with any mammalian protein (Hanner et al., 1996). It is widely distributed in peripheral organs and different areas of the central nervous system involved in memory, emotion, sensory and motor function (Wolfe et al., 1989; Brust et al., 2014). The generation of the Sigma-1 knockout mice in 2003 contributed to better understand the *in vivo* role of Sigma-1 receptors (Langa et al., 2003). The concept of the Sigma-1 receptor has evolved significantly over the past decades. Today, it seems clear that the Sigma-1 is not a traditional receptor. It is considered to be a non-G-protein coupled, non-ionotropic intracellular chaperone at the endoplasmic reticulum (ER) that modulates Ca^2+^-signaling (Hayashi and Su, 2007; Kim, 2017; Penke et al., 2018). Nevertheless, we are only just beginning to understand what the Sigma-1 protein does and how it works.

The most popular methods to study the function of a specific protein mainly include molecular, cellular and pharmacological approaches, and bioinformatic analysis. However, very few researchers utilize systematic bibliometric analytical approaches to study a specific protein or gene. Bibliometric analysis is a widely used quantitative method to examine the knowledge structure and development in research fields (Portillo-Salido, 2010; Guler et al., 2016; Munoz-Ecija et al., 2017). It is widely used in various areas to estimate the productivity of institutions, countries, and authors; and identify international collaborations and geographic distributions (Chinchilla-Rodríguez et al., 2015). More recently it is being used to explore research hotspots and frontiers in specific fields such as diseases (Zhou et al., 2018), materials (Vargas-Quesada et al., 2017), genes/proteins/targets (Zongyi et al., 2016; Lu et al., 2018; Zhao et al., 2018), and drugs (Sweileh et al., 2016; Hernandez-Vasquez et al., 2018; Zyoud et al., 2018). To our knowledge, no previous bibliometric analysis on Sigma-1 receptor has been published. When searching for the term “sigma-1 receptor” in the Scopus or Pubmed scientific databases, the first two references that appear are from Basile et al. (1992a,b). Previously, the more general term “sigma receptor” was used for many years but the use of this generic term progressively declined when, 4 years after Basile's articles, the Sigma-1 receptor was cloned. In the current study, we performed a bibliometric analysis to qualitatively and quantitatively specifically evaluate the Sigma-1 receptor studies until 2017. We take advantage of new visualization techniques based on bibliometric analysis of scientific publications to better approach the research focused on Sigma-1 receptor in the last 25 years. Our objectives were to describe the scientific outputs of Sigma-1 receptor research and identify trends and hotspots. The main questions that became our guidelines for analysis were:

1. How vast and varied is Sigma-1 receptor research output?
2. What are the main research areas in which the role of Sigma-1 receptor has been explored?
3. What were the most influential authors and publications in Sigma-1 receptor research?
4. What was the level of collaboration between the Sigma-1 receptor research community?

## Methods

### Study Design

A bibliometric analysis using documents published until December 2017 in journals indexed in Scopus (https://www.scopus.com/) was performed. While there are a variety of document types, only articles were included.

### Source of Information

Scopus (Elsevier BV Company, USA) is the largest abstract and citation database of scientific peer-review literature, including more than 22,000 titles from international publishers. We decided to use this database because it includes all MEDLINE documents and other characteristics, such as country of all authors and citations per document—this information being relevant to this study (Falagas et al., 2008; Kulkarni et al., 2009; Agarwal et al., 2016).

### Search Strategy

A literature search was conducted by the authors in Scopus for publications on a single day, May 15, 2018, and used the following search: [TITLE-ABS-KEY (“Sigma-1 receptor”) OR TITLE-ABS-KEY (“SigmaR1”) OR TITLE-ABS-KEY (“Sigma type 1”) OR TITLE-ABS-KEY (“Sig1r”) OR TITLE-ABS-KEY (“Sigma1 receptor”) OR TITLE-ABS-KEY (“Sigma-1 agonist”) OR TITLE-ABS-KEY (“Sigma-1 antagonist”) OR TITLE-ABS-KEY (“Sigma-1 ligand”) OR TITLE-ABS-KEY (“Sigma1 agonist”) OR TITLE-ABS-KEY (“Sigma1 antagonist”) OR TITLE-ABS-KEY (“Sigma1 ligand”) OR TITLE-ABS-KEY (“Sigma1-binding”)] AND DOCTYPE (ar) AND PUBYEAR < 2018 AND [LIMIT-TO (LANGUAGE, “English”)]. The validity of the search strategy was tested by manually reviewing the retrieved articles.

### Data Analysis

All data were collected by the authors and downloaded in csv format. The data were imported to Microsoft Excel 2013 and quantitatively and qualitatively analyzed. Some data had to be standardized because documents mistakenly attributed to the domain of author name and affiliation were detected. Therefore, standardization was carried out manually by the authors. Different outputs were extracted from Scopus, including annual research, countries, journals, authors, institutions, and citation frequency. The annual publications and average citations per year per publication, the relationship between the average number of times cited per paper and the number of years since its publication was calculated. The mean number of citations per publication (CPP), including article lifespan for all 1,102 articles and for those with more than 150 citations was also analyzed. For journal analysis we used Bradford's law as a bibliometric indicator for the dispersion of scientific information. This law first described by Samuel C. Bradford in 1934 is to show the distribution of the scientific literature in a particular discipline, and Bradford proposed a model of concentric zones of productivity (Bradford zones) with decreasing density of information that can be used to identify the ”core“ journals in a field (Brookes, 1969; Desai et al., 2018). One formulation is that if journals in a field are sorted by the number of articles into three zones, each with approximately one-third of all articles, then the number of journals in each zone will be proportional to 1:n:n2. We also summarized the number of journal articles and percentage of total, cumulative number of articles published by the journals and percentage of Sigma-1 receptor articles, SCImago Journal Rank (SJR), CiteScore, best quartile, and categories.

To analyse the impact factor of the journals we used CiteScore and SCImago Journal Rank (SJR) from Scopus. CiteScore is a new journal metric recently launched by Elsevier which is similar to the Journal Impact Factor (Journal Citations Reports, Clarivate Analytics). CiteScore is the number of citations received by a journal in 1 year to documents published in the 3 previous years, divided by the number of documents indexed in Scopus published in those same 3 years (see https://service.elsevier.com/app/answers/detail/a_id/14880/supporthub/scopus/ for details). SCImago Journal Rank indicator expresses the average number of weighted citations received in the selected year by the documents published in the selected journal in the three previous years. Citation weighting depends on subject field and prestige of the citing serial (see https://www.scimagojr.com/ for further details). The contributions of countries were evaluated based on paper and citation numbers, and the research output of each country was adjusted according to population size (http://www.worldbank.org/). For author and cited reference analysis, the top 15 productive and cited authors on Sigma-1 receptor research, along with their h-index, period of activity, and number and citations per article was analyzed. The research areas (e.g., Neuroscience, Medicine, Chemistry, etc.) were defined as described in SCOPUS.

### Visualization Maps

Several visualization tools for bibliometrics have been developed and are now frequently used, including Vosviewer, Citespace, Bicomb, and BibExcel (Chen, 2004). These tools have been developed to help researchers create knowledge maps, evaluate the collective state of the art about a subject, and identify hotspots in a research field. The two most common methods are co-occurrence and co-citation analysis. Co-occurrence analysis helps researchers identify the hot topics and trends in a discipline. If two words co-occur frequently in an article, they may have a closer relationship than other pairs of words. The citations in an article can provide important insight into what is currently known about a given topic. The most common method to acquire this information is by co-citation analysis: two papers that are both cited by a third article have a co-citation relationship. The strength of this relationship between articles can help researchers identify the intellectual base of the discipline, research frontiers, important authors, and other relevant bibliometric information (Chen, 2004; Vargas-Quesada et al., 2017).

We used VOSviewer version 1.6.9 for viewing and creating the desired bibliometric maps (http://www.vosviewer.com/; Leiden University, Netherlands; van Eck and Waltman, 2010). It is a software tool for building and depicting networks based on bibliometric data. It features a text mining instrument that can be used to depict co-occurrence networks of terms extracted from any part of scientific literature. The terms maps were used to explore trends and active growth areas. To explore the knowledge structure and main lines of Sigma-1 receptor research, we selected Author Keywords as the unit of analysis; their co-occurrence was, as we mentioned before, the unit of measurement (full counting). When two different keywords were used to define the same concept, normalization was applied (for instance the different variants used for the term “positron emission tomography” were normalized to “PET”). Country co-authorship, author co-authorship and author co-citation were also presented as network visualization maps. In VOSviewer maps, the size of the label and the circle of an item are determined by the weight of the item. The higher its weight, the larger its label, and circle. The color of an item is determined by the cluster to which the item belongs. Lines between items represent links. By default, at most 1,000 lines are displayed and represent the 1,000 strongest links between items. The distance between two items in the visualization approximately indicates the relatedness of the items in terms of co-authorship, co-occurrence, citation, bibliographic coupling, or co-citation links.

### Research Ethics

The data were downloaded from Scopus; these being secondary data, no interaction with animal or human subjects was involved. There were no ethical questions about the data. Approval by an ethics committee was not necessary.

## Results and Discussion

### Analysis of Publication Outputs and Citations

**Figure 2:**
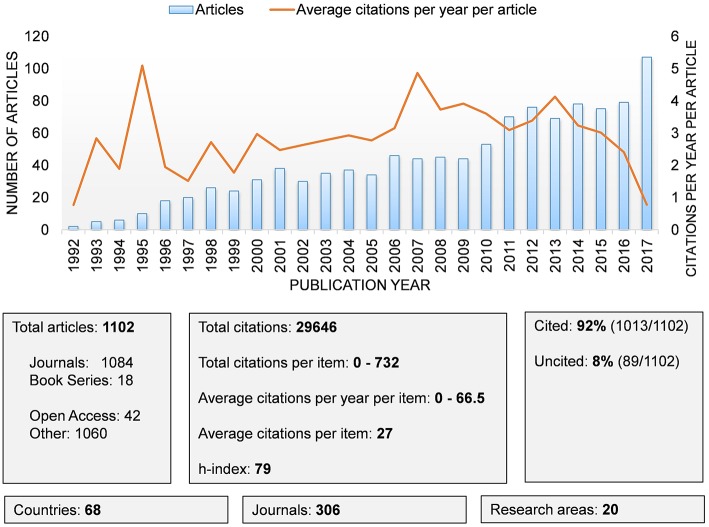
Evolution of scientific publications in the field of Sigma-1 receptor research.

Annual publications and average citations per year per publication on the Sigma-1 receptor are summarized in Figure 2. From 1992 to 2017, there were 1,102 publications on the Sigma-1 receptor, including 1,084 articles published in Journals and 18 Book Series. While the annual number of publications increased over time, growth rate fluctuations were observed. Thus, the distribution of publications can be divided into different time stages. Sigma-1 receptor research was initiated in 1992–1995, with increased research in 1996–2001; twice as many publications were found in 2001 (38 articles) vs. 1996 (18 articles). As compared to the past 5 years (1996–2001), the publication growth rate suddenly decreased in the period of 2001–2009 (1.2-fold). From 2009 to 2012 the growth rate partially recovered again (1.73-fold). Finally, there was no growth in the number of articles from 2012 to 2016. Interestingly, the last year analyzed (2017) reached a peak of 107 publications, which represent the largest increase in the number of articles with respect to previous year. The sum of all citation numbers is 29,646. Thus, the average citation value was 27 per paper and the h-index was 79. A total of 3,531 authors in 2,697 organizations from 68 countries were found. When analyzing the average number of citations per year per published item since 1992, the 10 articles published in 1995 and the 44 articles published in 2007 reached peaks of 5.09 and 4.86 citations per year, respectively. These citation peaks were due to three articles that were frequently cited: (Monnet et al., 1995) (16.35); (Vilner et al., 1995) (16.26); (Hayashi and Su, 2007) (66.55) (discussed below).

**Figure 3:**
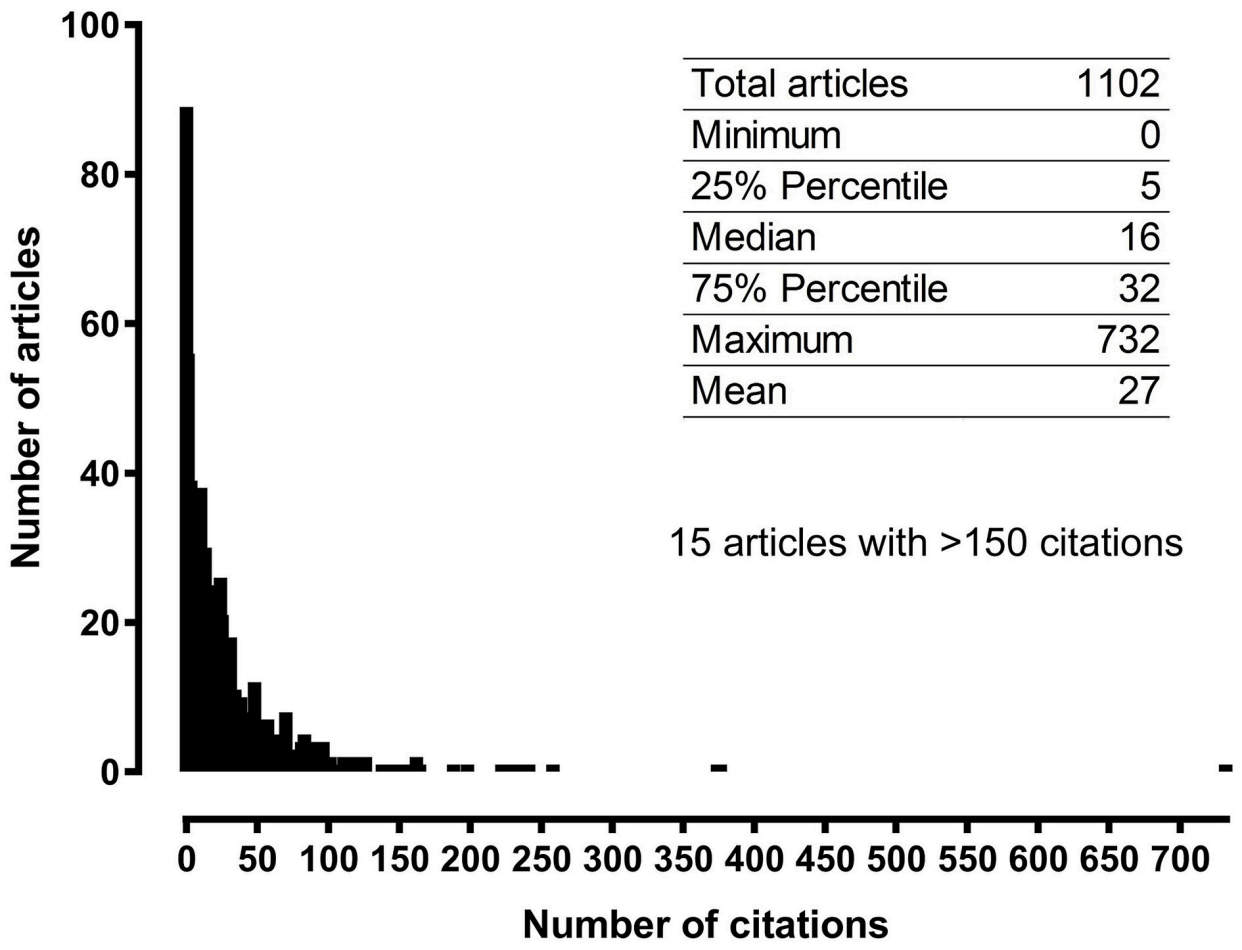
Frequency histogram showing the number of citations of Sigma-1 receptor articles published between 1992 and 2017.

Figure 3 shows the histogram of the citation data for all Sigma-1 receptor papers. Among all 1,102 Sigma-1 related articles in the pool indexed in the Scopus publication database, 89 papers (~8% of the pool) had no citations at all, 86% received fewer than 50 citations, and 75% received fewer than 32 citations. The median research paper on the Sigma-1 receptor received 16 citations, and only 15 articles received more than 150 citations. The most highly cited paper was cited 732 times (discussed below).

**Figure 4:**
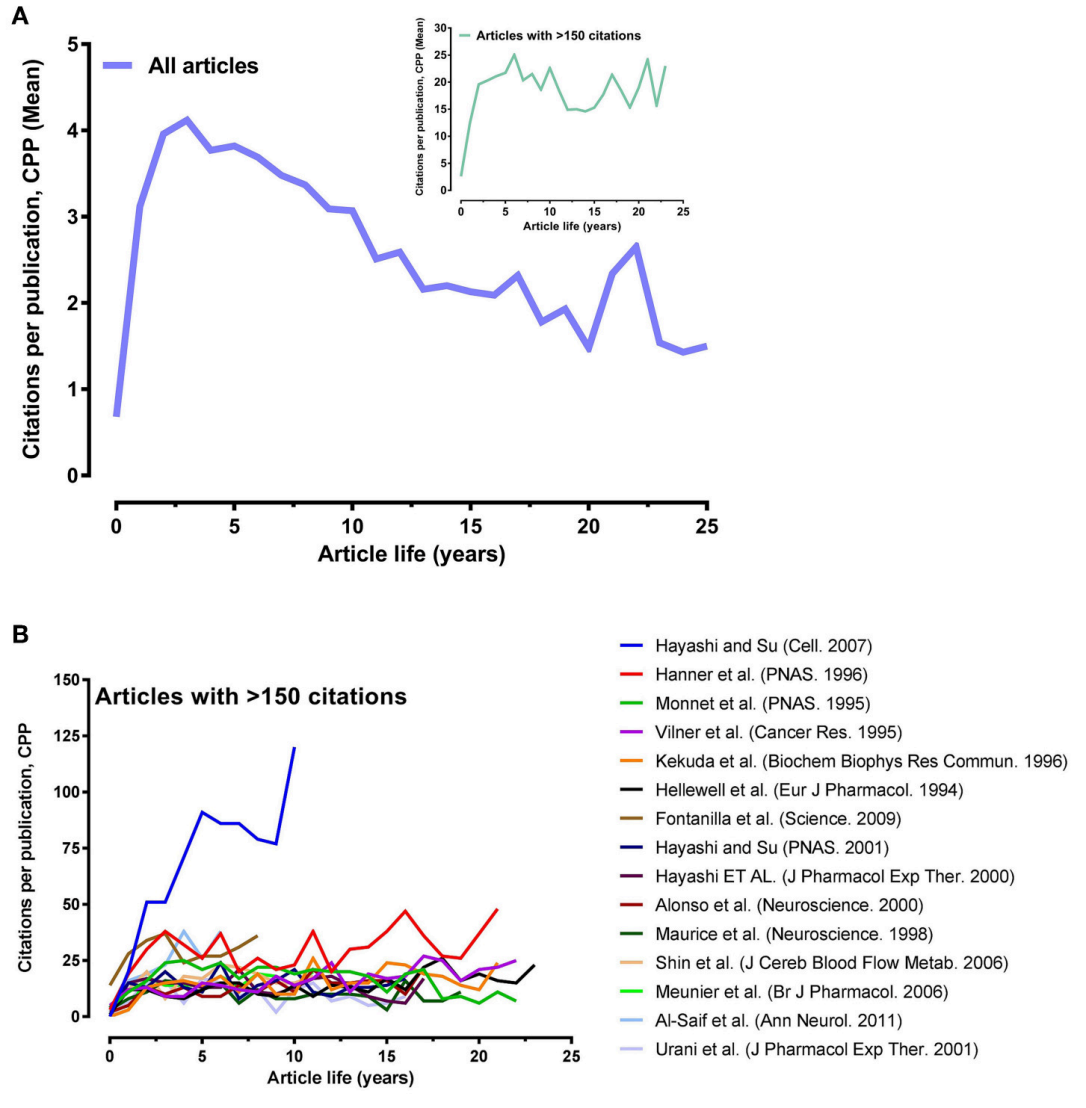
Variations of citations per publication (CPP) with article lifespan. **(A)** Mean CPP with article lifespan for all 1102 articles and for those with more than 150 citations (insert graph). **(B)** CPP values of each highly cited article over the years.

The citation of an article usually follows a time course. The article lifespan demonstrates the influence of the article on scientific research. The impact of normal publications increases during the first years after publication, peaks after 3–5 years and then decreases over time (Costas et al., 2011). Figure 4 shows the citation pattern of the Sigma-1 receptor publications, i.e., the relationship between the average number of times cited per paper and the number of years since its publication. Figure 4A shows the mean number of citations per publication (CPP), including article lifespan for all 1102 articles and for those with more than 150 citations (insert graph). CPP values for all 1102 articles significantly increased over the first 2 years, peaked in the 3rd year (CPP ~4) and declined thereafter. Only 15 papers received more than 150 citations. These articles were published between 1995 and 2011. CPP values from these highly cited articles also significantly increased over the first 2 years but peaked only in the 6th year (CPP ~25). The mean number of citations for the 15 most cited articles was ~261 times.

Citation frequency curves of individual articles can exhibit one of the following patterns: 1) initially much praised work, 2) basic recognized work, 3) scarcely reflected work, 4) well-received but later erroneous qualified work, and 5) genius work (Avramescu, 1979). Figure 4B illustrates the CPP values of each highly cited article over the years. Two types of citing patterns can be observed, including basic recognized work and one genius work. The article published by Hayashi and Su (2007), the genius work, stands out prominently. It is the most highly cited recent article and does not seem to have reached its peak yet, as evidenced by the steady CPP value increase over the years. The other 14 highly cited articles had lower CPP values and remained essentially constant.

### Journal Analysis

More than 300 scholarly journals have published articles on Sigma-1 research. Bradford's law of scattering is a pattern first described by Samuel C. Bradford in 1934 that estimates the exponentially diminishing returns of extending a search for references in science journals, and that can be used to identify the ”core“ journals in a field (Brookes, 1969; Desai et al., 2018). One formulation is that if journals in a field are sorted by the number of articles into three zones, each with approximately one-third of all articles, then the number of journals in each zone will be proportional to 1:n:n^2^. Table 1 shows the Bradford zones of scattering for Sigma-1 receptor literature. Our sample from 1992 to 2017 includes 306 journals. Of these, 170 journals have published only 1 paper on the Sigma-1 receptor. Table 1 lists the nucleus and the successive zones of journals. Three zones, each publishing approximately 33% (367 articles) of the total Sigma-1 receptor articles (1,102 articles), constitute the most specific subdivisions. We found that 3.6% (11 journals) of the journals that published articles on Sigma-1 research were distributed in zone 1, 14% (44 journals) were distributed in zone 2, and 82% (251 journals) were distributed in zone 3, which had a lower influence than zone 1 or 2 (Table 1).

**Table 1:** Bradford's Law of Scattering for journals that published articles on Sigma-1 receptor research from 1992 to 2017.

	***n***	***n/N (%)***
Zone 1	11	3.6
Zone 2	44	14.4
Zone 3	251	82.0

*Each zone represents about 33% of the total articles (1,102); n, the number of journals in each zone; N, the number of all journals (306)*.

To more closely examine the leading journals, Table 2 lists the number of journal articles in descending order and percentage of total, cumulative number of articles published by the journal from 1992 to 2017 and percentage of Sigma-1 receptor articles, CiteScore, SCImago Journal Rank (SJR), best journal quartile, and categories. We used CiteScore and SJR from Scopus as an indicator of the publication's repercussion. As indicated in Table 2, the core Sigma-1 receptor literature concentrates on a small number of Pharmacology, Drug Discovery and Chemistry related journals. Others categories are Radiology Nuclear Medicine and Imaging, Neurosciences, Biochemistry, and Biology. Out of the top 15 journals analyzed, the European Journal of Pharmacology (CiteScore2017: 3.18, SJR2017: 1.06, 73 articles, 7%) ranks first in the number of Sigma-1 receptor publications, followed by the Journal of Medicinal Chemistry (CiteScore2017: 6.25, SJR2017: 2.75, 56 articles, 5%) and the Journal of Pharmacology and Experimental Therapeutics (CiteScore2017: 3.70, SJR: 1.59, 47 articles, 4%). These three journals published 16% of the total articles. Additionally, Nuclear Medicine and Biology (CiteScore2017: 2.12; SJR2017: 0.70) devoted 24 articles (0.8% of its publications) to Sigma-1 research, followed by Synapse (CiteScore2017: 2.18; SJR2017: 0.97, 23 articles, 0.7% of its publications). As compared to other journals, articles on Sigma-1 research were more likely to be accepted by these active journals. The 15 most active journals (80% in Quartile 1; 13% in Quartile 2; 7% in Quartile 3) published approximately the 40% of the Sigma-1 receptor articles.

**Table 2:** The 15 most active journals that published articles on Sigma-1 receptor research from 1992 to 2017.

**Journal**	**Sigma-1 articles**	**%**	**Total articles in the journal**	**%**	**CiteScore 2017**	**SJR 2017**	**Best quartile**	**Categories**
Eur. J. Pharmacol.	73	7	24,529	0.3	3.18	1.06	Q1	Pharmacology
J. Med. Chem.	56	5	22,665	0.2	6.25	2.57	Q1	Drug Discovery; Molecular Medicine
J. Pharmacol. Exp. Ther.	47	4	22,048	0.2	3.70	1.59	Q1	Pharmacology; Molecular Medicine
Bioorg. Med. Chem.	38	3	13,358	0.3	2.90	0.87	Q1	Pharmaceutical Science; Organic Chemistry
Bioorg. Med. Chem. Lett.	25	2	25,609	0.1	2.53	0.81	Q1	Pharmaceutical Science
Neuropharmacology	24	2	9,520	0.3	4.65	2.04	Q1	Pharmacology; Cellular and Molecular Neuroscience
Nucl. Med. Biol.	24	2	3,024	0.8	2.12	0.70	Q1	Radiology Nuclear Medicine and imaging
Br. J. Pharmacol.	24	2	20,003	0.1	5.97	2.60	Q1	Pharmacology
Synapse	23	2	3,300	0.7	2.18	0.97	Q3	Cellular and Molecular Neuroscience
Psychopharmacology	17	2	12,924	0.1	3.05	1.49	Q2	Pharmacology
Pharmacol. Biochem. Behav.	17	2	12,227	0.1	3.02	1.15	Q1	Behavioral Neuroscience
Eur. J. Med. Chem.	17	2	9,381	0.2	4.63	1.27	Q1	Pharmacology; Organic Chemistry; Drug Discovery
Brain Res.	16	1	51,636	0.03	3.02	1.40	Q1	Clinical Neurology
PLoS ONE	16	1	183,064	0.01	3.01	1.16	Q1	General Agricultural and Biological Sciences; General Biochemistry, Genetics and Molecular Biology
Adv. Exp. Med. Biol.	15	1	7,168	0.2	1.67	0.87	Q2	General Biochemistry, Genetics and Molecular Biology

All 15 most active journals have a CiteScore ranging from 1.67 (Advances in Experimental Medicine and Biology) to 6.25 (Journal of Medicinal Chemistry). The top journals with a CiteScore > 5 (2/15; 13% of the top journals) published 7% of the total number of Sigma-1-related articles. The top journals with CiteScore 3–5 (8/15; 53% of the top journals) published 21% of the total number of Sigma-1-related articles. The top journals with CiteScore < 3 (5/15; 33% of the top journals) published 10% of the total number of Sigma-1-related articles. In summary, when comparing the rate of Sigma-1 receptor articles medium-CiteScore journals (CiteScore 3–5) to that of all journals (rate of journals with CiteScore > 10, 0.7%; CiteScore 5–10, 3.1%; CiteScore 3–5, 8.8%; and CiteScore < 3, 87.4%) (https://www.scopus.com/sources), Sigma-1 receptor articles were relatively intensively published in medium CiteScore journals.

### Country, Institution, and International Collaboration Analysis

The top countries, country institutions and institution authors with more publications on Sigma-1 receptor research from 1992 to 2017 are shown in Table 3. Sigma-1 receptor publications were produced by countries from different world geographies, including countries outside Europe and North America. The United States was the country with the highest production with 405 (37%) documents, followed by Japan and Germany with 228 (21%) and 103 (9%) documents, respectively. Spain (1.8), Japan (1.8) and Italy (1.6) showed the highest per million inhabitants ratio. The United States had the highest h-index of 63, followed by Japan (h-index 43) and France (h-index 40). The highest citation/article ratio was reached in France (56), United States (34) and Canada (33). The leading institutions were the University of Münster (67; 65% of Germany documents), the National Institutes of Health (65; 16% of The United States documents), ESTEVE (48; 58% of Spain documents) and INSERM (45; 54% of France documents). The main research area within these four institutions was related to chemistry, drug addiction, pain, and cognition/neuroprotection, respectively.

**Table 3:** Top countries, country institutions and institution authors with more publications on Sigma-1 receptor research from 1992 to 2017.

**Country**	**Articles**	**Citations**	**h-Index**	**Citations per article**	**Articles per million inhabitants**	**Top country institution (top institution author)**	**Institution articles**	**%**	**Main research area of the institution in Sigma-1 field**
United States	405	13,634	63	34	1.2	National Institutes of Health (Su, T.P.)	65	16	Drug addiction
Japan	228	5,660	43	25	1.8	Tokyo Metropolitan Institute of Gerontology (Ishiwata, K.)	35	15	Neuroimaging/PET
Germany	103	937	27	9	1.2	University of Münster (Wünsch, B.)	67	65	Chemistry
Italy	98	1,944	25	20	1.6	University of Catania (Prezzavento, O.)	29	30	Chemistry; Neuroprotection; Memory; Pain, …
China	85	1,009	20	12	0.1	Nanjing Medical University (Chen, L.)	23	27	Neuropsychiatric disorders; Neuroprotection, …
France	84	4,719	40	56	1.3	INSERM (Maurice, T.)	45	54	Cognition; Neuroprotection
Spain	83	1,877	28	23	1.8	ESTEVE (Vela, J.M.)	48	58	Pain
South Korea	33	727	18	22	0.6	Seoul National University (Roh, D.H. & Yoon, S.Y.)	21	64	Pain
United Kingdom	33	882	19	27	0.5	University of East Anglia (Duncan, G. & Wang, L.)	5	15	Eye
						University of Cambridge (Balasuriya, D. & Edwardson, J.M.)	5	15	Neurobiology; Protein-proten interaction
Australia	27	477	13	18	1.1	University of Sydney (Kassiou, M.)	12	44	Chemistry
Canada	22	722	15	33	0.6	McGill University (Debonnel, G.)	8	36	Depression
Poland	22	414	12	19	0.6	Polish Academy of Sciences (Skuza, G.)	17	77	Depression

Nineteen companies published 119 articles (11% of the total articles) (Table 4). Out of 19 companies publishing about the Sigma-1 receptor, 2 companies contributed 63% of the papers. The most productive company was ESTEVE (now Esteve Pharmaceuticals) with 48 publications, followed by Santen Pharmaceutical, which contributed 27 articles until 2004, thus suggesting that they are not currently active in Sigma-1 receptor research. Santen Pharmaceutical is the originator company of Cutamesine (SA-4503), a Sigma-1 receptor denoted as agonist/activator that was under development for the treatment of amyotrophic lateral sclerosis, age-related macular degeneration, acute ischemic stroke, major depressive disorder, traumatic brain injury, multiple sclerosis, stroke, and retinitis pigmentosa. While the drug was tested in phase II in patients with major depressive disorders and in patients recovering from stroke, its development was terminated for the given conditions. Currently, M's science corporation (under license from Santen) is supposedly developing cutamesine for the potential treatment of amyotrophic lateral sclerosis and retinitis pigmentosa as more suitable target diseases (Source: https://pharma.globaldata.com/). Esteve Pharmaceuticals is currently active in the development of Sigma-1 receptor antagonists/inhibitors for the treatment of neuropathic pain (Source: https://www.esteve.com/en/research-development).

**Table 4:** Companies that publish on Sigma-1 receptor research.

**Company**	**Country**	**Total publications**	**Corresponding publications**	**Publication years**
ESTEVE	Spain	48	26	1999–2017
Santen Pharmaceutical	Japan	27	13	1997–2004
Hamamatsu Photonics	Japan	7	1	2003–2017
Sanofi-Synthelabo	France	6	4	1997–2003
Mitsui Pharmaceuticals	Japan	4	2	1999–2001
Nihon Schering (formerly Mitsui)	Japan	4	4	2001–2002
Taisho Pharmaceutical	Japan	4	4	1999–2004
UCB Pharma /UCB Research	Belgium/United States	3	2	2004–2013
Nensius Research	Denmark	2	2	2011–2013
Teva Pharmaceutical Industries	Israel	2	2	2016–2017
Merck Research Laboratories	United States	2	1	1994–2007
NitroMed	United States	2	1	1999–2004
Anavex Life Sciences	Greece	2	0	2007–2013
Servier	France	1	1	1998
Newron Pharmaceuticals	Italy	1	1	2000
BioNeuroFar	Italy	1	1	2007
PerkinElmer Health Sciences	United States	1	1	2012
Daya Drug Discoveries	United States	1	1	2014
Corden Pharma Switzerland	Switzerland	1	1	2016

**Figure 5:**
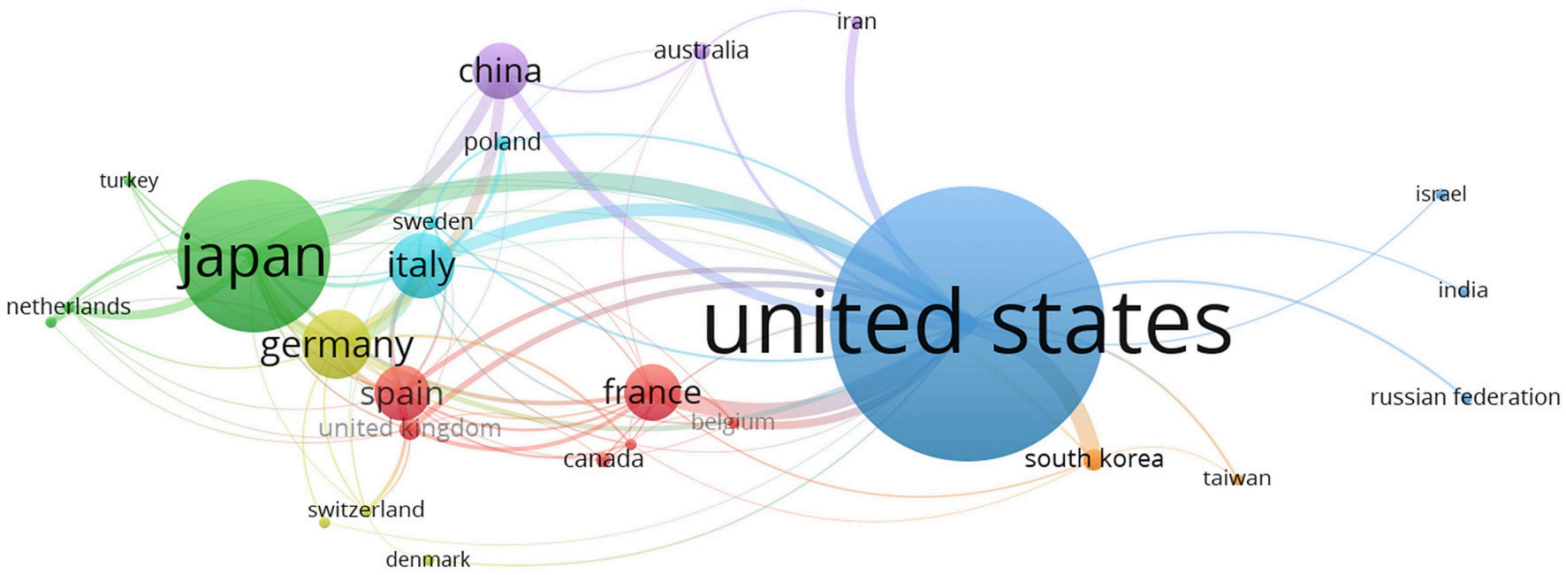
VOSviewer network visualization map of country co-authorship (international collaboration) for Sigma-1 receptor publications, 1992–2017. Twenty-six out of the 68 countries had at least 5 publications; the largest set of connected countries consists of 26 countries in 7 clusters.

We used VOSviewer to visualize the network map of country co-authorship (international collaboration) for Sigma-1 receptor publications. Distribution maps provide valuable information and help researchers identify potential collaborators. The largest set of connected countries consists of 26 countries in 7 clusters. Figure 5 illustrates the collaborative network of countries publishing more than five documents (26 of the 68 countries). Clusters are formed by the frequency of co-occurring terms representing each country, the more often the terms tend to co-occur they get colored into clusters. The size of circles represents the number of publications of the country and the thickness of lines depicts the size of collaboration. For example, the link strength (collaboration) between the United States and Japan was 20 and it represents a thick line. On the other hand, the line between the United States and India had a link strength of 2. Countries with similar color form one cluster. The blue cluster shows collaborative links between the largest circles of the United States, Japan (green color) and Italy representing authors affiliated to these countries. The United States collaborated the most with other countries worldwide. Other international researchers who collaborated the most with the United States researchers were from South Korea, France, Italy, and China. Spain is associated with France (red color), and to a lesser extent other countries such as the United Kingdom and Canada. The purple cluster is leaded by China collaborating with Japan and the United States.

### Author and Cited Reference Analysis

The 1,102 articles on the Sigma-1 receptor were drafted by more than 3,000 authors. Table 5 presents the top 15 productive and cited authors on Sigma-1 receptor research, along with their h-index, period of activity, and number and citations per article. Table 6 shows the 15 most frequently cited references. Eleven out of the 15 most frequently cited articles were published in journals with a high impact factor (CiteScore > 7). Hayashi-T and Su-TP (Cell. 2007) is the top cited reference (732 citations, 66.5 citations per year). Each of the 15 most active authors contributed at least 22 articles to Sigma-1 research. Wünsch (73 articles), who mainly participated in chemistry-related papers, ranks first, followed by Schepmann-D (57 articles), who is a coauthor in the Wünsch's articles, and by Maurice-T (45 articles), who mainly focused on the role and applications of Sigma-1 receptor and ligands to cognitive/behavioral diseases. Most of the productive authors were also the most cited ones, with some particularities. Wünsch, who is the most productive, ranks thirteenth in terms of citations per article. Most cited authors were Hayashi-T and Su-TP, who had the most cited publication in the field of the Sigma-1 receptor (Table 6 and Figure 4B). Their paper identified the Sigma-1 receptor as a novel “ligand-operated” chaperone and characterized the important role played by the Sigma-1 receptor in endoplasmic reticulum-mitochondrial interorganelle Ca^2+^ signaling and also in cell survival. These two authors have two more articles among the most cited ones (Hayashi and Su, 2001; Kourrich et al., 2013; Table 6) which demonstrated that Sigma-1 receptor modulation of different proteins contribute to the effects of cocaine, neurosteroids and other drugs by regulating intracellular Ca^2+^ signaling. The third most cited author is Bowen-WD, who ranks twelfth in number of articles, followed by Maurice-T, who also ranks third in number of articles. Maurice-T also scores first in h-index (h-index = 30). As shown in Table 6, Bowen-WD contributed an early article, which is among the most cited ones, describing the expression of Sigma-1 and Sigma-2 receptors in a wide variety of human and rodent tumor cell lines (Vilner et al., 1995). A paper authored by Maurice-T describing the involvement of the Sigma-1 receptor in the anti-amnesic and neuroprotective effects of donepezil in a mouse model of Alzheimer's disease in mice is also among the most cited (Meunier et al., 2006) (Table 6). The paper by Schmidt et al. (2016), published in Nature and reporting the crystal structure of the human σ1 receptor, ranks second and is followed by a paper in Science by Fontanilla et al. (2009) in terms of average citations per year. Schmidt-HR showed the overall architecture, oligomerization state and molecular basis for ligand recognition of the Sigma-1 receptor, and Fontanilla-D described N,N-dimethyltryptamine (DMT), which has shown hallucinogenic properties, as an endogenous Sigma-1 receptor regulator. Other highly cited articles on Sigma-1 focused on the role of Sigma-1 in a number of diseases with important medical needs such as Alzheimer's disease (Hedskog et al., 2013), amyotrophic lateral sclerosis (Al-Saif et al., 2011), cancer (Vilner et al., 1995), and Parkinson disease (Francardo et al., 2014). Finally, two articles on Sigma-1 receptor cloning and expression (Hanner et al., 1996; Kekuda et al., 1996) and one on Sigma-1 and Sigma-2 receptor characterization (Hellewell et al., 1994) also appear as highly cited in Table 6.

**Table 5:** The 15 most active authors in Sigma-1 receptor research from 1992 to 2017.

**Author**	**Articles**	**Document h-index**	**Citations**	**Citations per article**	**Year of 1st-last Sigma-1 publication**
Wünsch, B.	73	23	1,267	17	2001–2017
Schepmann, D.	57	19	856	15	2006–2017
Maurice, T.	45	30	2,642	59	1996–2016
Ishiwata, K.	36	19	987	27	1998–2016
Su, T.P.	34	25	2,991	88	1998–2017
Mach, R.H.	29	16	780	27	1995–2016
Hayashi, T.	27	24	2,669	99	1995–2015
Ruoho, A.E.	27	17	1,013	38	2007–2017
Hashimoto, K.	26	14	777	30	1997–2003
Matsumoto, R.R.	26	15	632	24	1997–2017
Vela, J.M.	26	16	607	23	2003–2017
Bowen, W.D.	25	20	1,553	62	1993–2016
Matsuno, K.	25	19	1,086	43	1995–2004
Brust, P.	24	13	344	14	2008–2017
Zamanillo, D.	22	16	741	34	2000–2016

**Table 6:** The 15 most frequently cited references in Sigma-1 receptor research from 1992 to 2017.

**References**	**Average citations per year**	**Total citations**	**Title**	**Journal**	**CiteScore 2017**	**SJR 2017**
Hayashi and Su, 2007	66.5	732	Sigma-1 Receptor Chaperones at the ER- Mitochondrion Interface Regulate Ca^2+^ Signaling and Cell Survival	Cell. 2007 Nov 2;131(3):596–610.	21.99	25.14
Schmidt et al., 2016	35.0	70	Crystal structure of the human σ1 receptor	Nature. 2016 Apr 28;532(7600):527–30.	14.59	17.87
Hanner et al., 1996	29.7	653	Purification, molecular cloning, and expression of the mammalian sigma1-binding site	Proc Natl Acad Sci U S A. 1996 Jul 23;93(15):8072–7.	8.59	6.09
Fontanilla et al., 2009	28.7	258	The hallucinogen N,N-dimethyltryptamine (DMT) is an endogenous sigma-1 receptor regulator	Science. 2009 Feb 13;323(5916):934–7.	15.85	14.14
Hedskog et al., 2013	23.6	118	Modulation of the endoplasmic reticulum-mitochondria interface in Alzheimer's disease and related models	Proc Natl Acad Sci U S A. 2013 May 7;110(19):7916–21.	8.59	6.09
Al-Saif et al., 2011	23.1	162	A mutation in sigma-1 receptor causes juvenile amyotrophic lateral sclerosis	Ann Neurol. 2011 Dec;70(6):913–9.	7.62	5.71
Kourrich et al., 2013	16.8	84	Dynamic interaction between sigma-1 receptor and Kv1.2 shapes neuronal and behavioral responses to cocaine	Cell. 2013 Jan 17;152(1–2):236–47.	21.99	25.14
Monnet et al., 1995	16.3	376	Neurosteroids, via σ receptors, modulate the [3H]norepinephrine release evoked by N-methyl-D-aspartate in the rat hippocampus	Proc Natl Acad Sci U S A. 1995 Apr 25;92(9):3774–8.	8.59	6.09
Vilner et al., 1995	16.3	374	Sigma-1 and Sigma-2 Receptors Are Expressed in a Wide Variety of Human and Rodent Tumor Cell Lines	Cancer Res. 1995 Jan 15;55(2):408–13.	7.35	4.26
Shin et al., 2006	15.7	188	Vasoconstrictive neurovascular coupling during focal ischemic depolarizations	J Cereb Blood Flow Metab. 2006 Aug;26(8):1018–30.	5.07	2.56
Kekuda et al., 1996	15.0	331	Cloning and functional expression of the human type 1 sigma receptor (hSigmaR1)	Biochem Biophys Res Commun. 1996 Dec 13;229(2):553–8.	2.62	1.09
Francardo et al., 2014	14.5	58	Pharmacological stimulation of sigma-1 receptors has neurorestorative effects in experimental parkinsonism	Brain. 2014 Jul;137(Pt 7):1998–2014.	7.43	5.86
Hayashi and Su, 2001	13.9	236	Regulating ankyrin dynamics: Roles of sigma-1 receptors	Proc Natl Acad Sci U S A. 2001 Jan 16;98(2):491–6.	8.59	6.09
Hellewell et al., 1994	13.8	330	Rat liver and kidney contain high densities of sigma 1 and sigma 2 receptors: characterization by ligand binding and photoaffinity labeling	Eur J Pharmacol. 1994 Jun 15;268(1):9–18.	3.18	1.06
Meunier et al., 2006	13.7	164	The anti-amnesic and neuroprotective effects of donepezil against amyloid B 25–35 peptide-induced toxicity in mice involve an interaction with the σ 1 receptor	Br J Pharmacol. 2006 Dec;149(8):998–1012.	5.97	2.60

**Figure 6:**
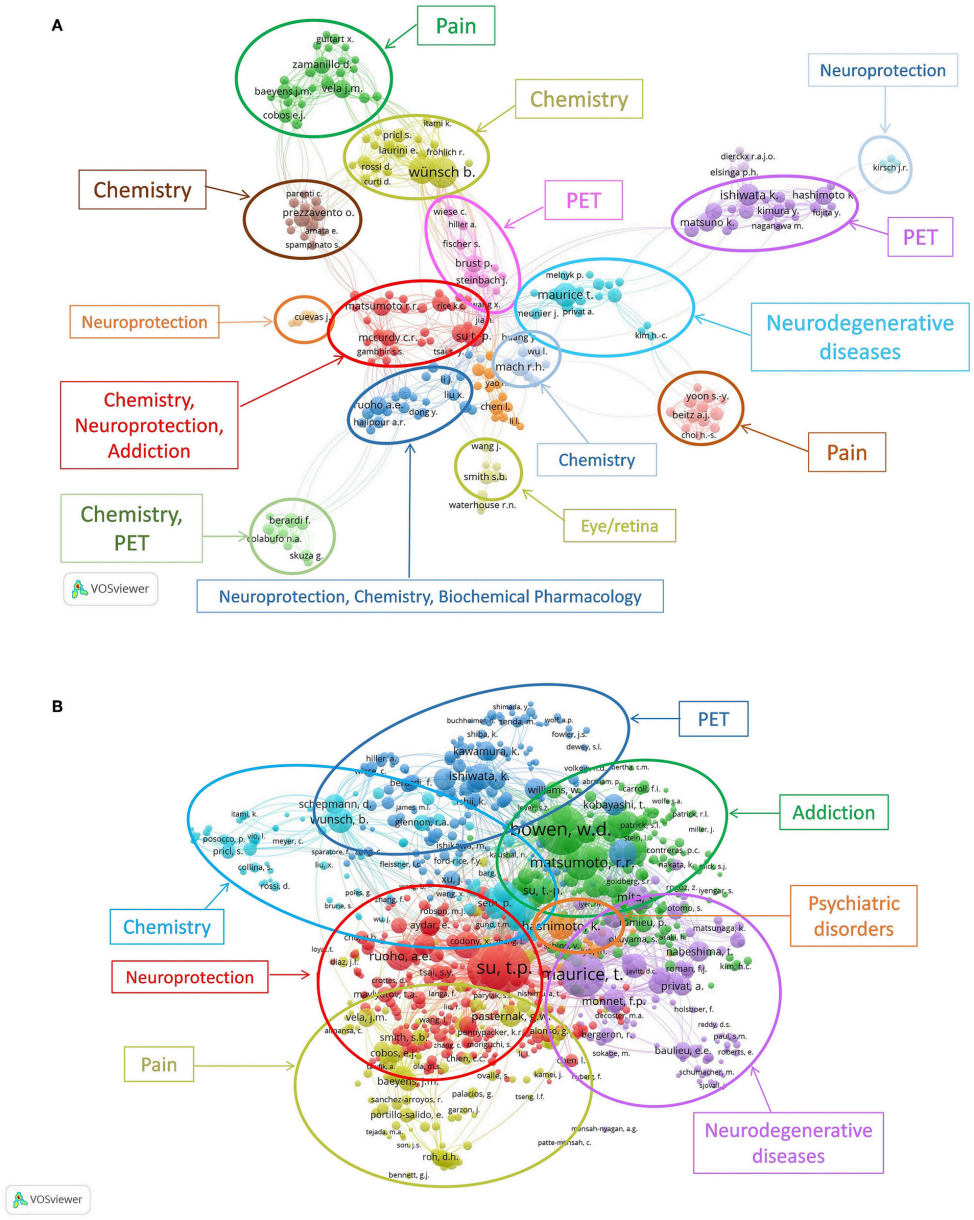
VOSviewer network visualization map of the authors for Sigma-1 receptor publications, 1992–2017. **(A)** Co-authorship analysis (the relatedness of items is determined based on the number of co-authored documents). Of the 3,529 authors, 247 had at least 5 publications; the largest set of connected authors consists of 224 authors in 16 clusters. **(B)** Co-citation analysis (the relatedness of items is determined based on the number of times they are cited together). Of the 51,564 authors, 1291 had at least 20 citations; the largest set of authors with greatest total link strength selected consists of 1000 authors in 7 clusters.

Citation networks have been applied to information science analysis. Here, VOSviewer was also used to analyse author citations in Sigma-1 research. VOSviewer is primarily intended to be used for bibliometric network analysis. The program can be used to create maps of publications, authors or journals based on a citation, co-citation, or bibliographic coupling network, or to create keyword maps based on a co-occurrence network. Two network visualization maps provided by VOSviewer are shown in Figure 6. In the co-authorship analysis (Figure 6A) the relatedness of items is determined based on the number of co-authored documents. Each node represents an author with at least five publications, the node label is the last name of the author, and the node size indicates the number of published articles. The link connecting two nodes stands for the cooperative relationship between two authors, and the thickness of the link stands for the intensity of cooperation. Another important information regarding authors is the degree of co-citation. Author co-citation analysis measures the number of times a particular group of authors was cited together within the collection (van Eck and Waltman, 2017). In the co-citation analysis (Figure 6B) the relatedness of items is determined based on the number of times they are cited together. Each node represents an author with at least 20 citations, the node label is the last name of the author, and edges represent citation relations. When co-authorship analysis was performed (Figure 6A), 247 out of the 3,529 authors had at least 5 publications, and the largest set of connected authors consists of 224 authors in 16 clusters. An inspection of the publications from each cluster allows displaying the main area for each research group. There are several independent clusters working in the same area. For instance, there are six clusters publishing articles on “Chemistry,” four clusters on “Neuroprotection,” three clusters on “PET,” two clusters on “Pain,” and one cluster related to those publishing mainly in “Addiction.”

The co-citation analysis by author included 51,564 cited authors, of which 1,291 were cited at least 20 times (Figure 6B); the largest set of authors with greatest total link strength selected consists of 1,000 authors in 7 clusters. In this case, each color represents a community of authors within the same subject of interest, and authors within a cluster represent a set of strongly connected authors in terms of co-citation relations. These clusters, as can be seen from Figure 6B relate to “Chemistry,” “PET,” “Neuroprotection,” “Addiction,” “Neurodegenerative diseases,” “Psychiatric disorders,” and “Pain.” These clusters point toward the pivotal scholars whose works were cited in the references of our collection and who contributed to build on each field studied (Figure 6B).

### Research Area Analysis

**Figure 7:**
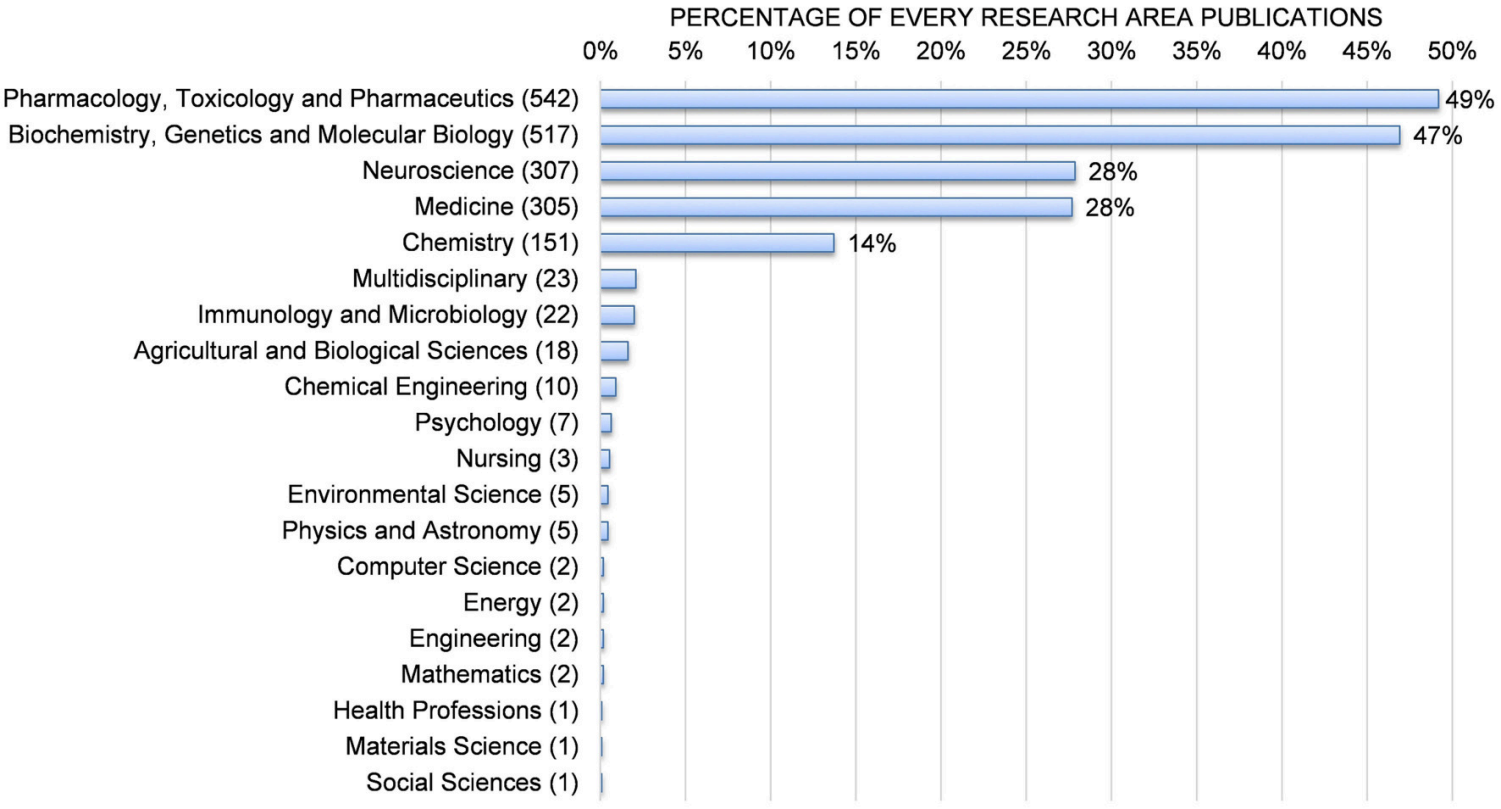
Sigma-1 receptor research areas from 1992 to 2017.

Research on the Sigma-1 receptor occurred in 20 special research areas. These 20 research areas, appearing in publications of Sigma-1 receptor research from 1992 to 2017, are shown in Figure 7. Here, the research areas (e.g., Neuroscience, Medicine, Chemistry, etc.) were defined as described in SCOPUS. “Pharmacology, Toxicology and Pharmaceutics” and “Biochemistry, Genetics, and Molecular Biology” accounted for the largest number of publications (49 and 47%, respectively), followed by “Neuroscience” (28%), “Medicine” (28%), and “Chemistry” (14%).

### Keyword Co-occurrence Cluster Analysis

The topics involved in Sigma-1 receptor research can be outlined in the keywords assigned to each article. Keywords have as their main objective to provide rapid access to scientific works and are highly effective in terms of bibliometric analysis when investigating the knowledge structure of scientific fields (Zhang et al., 2016; Vargas-Quesada et al., 2017). Keywords provide a reasonable description of research hotspots (attention by a number of scientific researchers focused on a set of related research problems and concepts).

**Figure 8:**
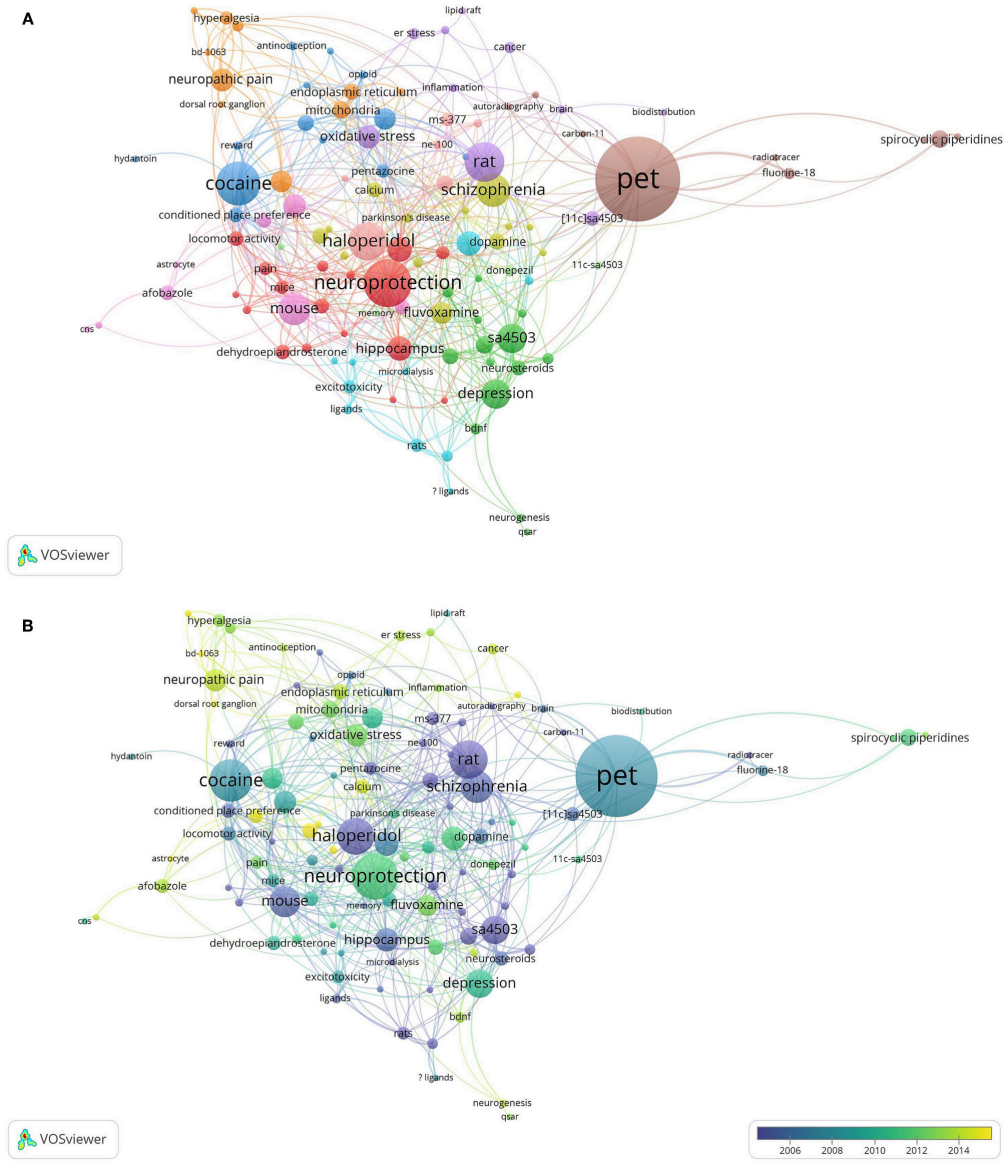
High-frequency terms in author keywords of Sigma-1 receptor publications during 1992–2017. Of the 2,247 keywords, 134 terms occurred at least 5 times. Omitting the term Sigma-1 receptor and the variants thereof, the largest set of connected keywords with greatest total link strength consists of 114 terms in 11 clusters. **(A)** VOSviewer network co-occurrence visualization map. **(B)** VOSviewer overlay visualization by time.

**Figure 9:**
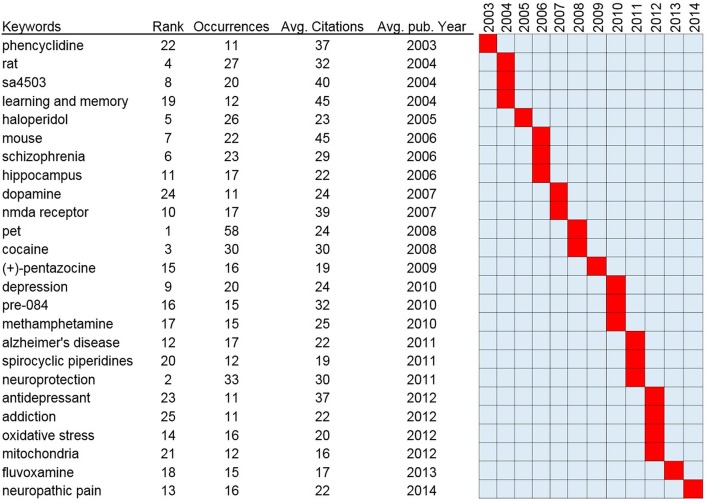
Top 25 keywords with strongest occurrences and average citations in articles related to Sigma-1 receptor research.

In the present study, VOSviewer was used to create a knowledge map of keyword co-occurrence with 114 terms in 11 clusters (Figure 8) and to identify the top 25 keywords in Sigma-1 research articles from 1992 to 2017, according to occurrences and citation counts, and ordered by average publication year (Figure 9). The top 5 keywords were “Positron Emission Tomography (PET),” “Neuroprotection,” “Cocaine,” “Rat,” and “Haloperidol” (Figure 9). High-frequency terms in author keywords such as “Learning and Memory” (2004), “Schizophrenia” (2006), “Positron Emission Tomography” (2008), “Cocaine” (2008), and “Depression” (2010) were found in the early years, as compared to terms such as “Alzheimer's disease” (2011), “Neuroprotection” (2011), “Addiction” (2012), and “Neuropathic Pain” (2014) in more recent years (Figure 8B).

## Conclusions

In this work, the global scientific outputs of Sigma-1 receptor research, its main research lines and its evolution are studied for the first time by means of bibliometric indicators of a basic nature and modern information visualization techniques. The resulting maps are a useful and attractive tool for the Sigma-1 receptor research community, as they reveal the main lines of exploration at a glance. The global view provided by our study shows that researchers have intended to explore the potential involvement of Sigma-1 receptors in some specific physiological processes and diseases. The hope that the modulation of the Sigma-1 receptor could be a therapeutic strategy is likely driving the Sigma-1 research community. Consistent with this, intensive activity has been carried out in the area of Medicinal Chemistry to obtain selective ligands that could be developed as drugs in the future. According to our analysis, the main diseases in which the Sigma-1 receptors have been explored include addiction, neuroprotection and neurodegenerative diseases, psychiatric disorders, and pain. Keyword co-occurrence analysis suggests that the research efforts made in some indications such as those in cocaine (addiction), learning and memory or depression/schizophrenia/ haloperidol (psychiatric conditions) have declined over time, while others such as those focusing on neuroprotection/Alzheimer's disease (neurodegenerative diseases) or pain are currently most popular. Early studies on psychiatric disorders, learning and memory or cocaine did not translate into marketed drugs, and hopes now seem to be placed on studies relating to neurodegenerative diseases and pain. Leaving aside the well-known low success in transforming basic research into new drugs with a clear therapeutic potential and difficulties related to drug discovery programs (Paul et al., 2010), the lack of selective Sigma-1 ligands approved for use in humans could be the result of insufficient research effort/interest by the scientific community, including biopharmaceutical companies. In fact, only 247 authors have five or more publications and the growth in the production of articles is not constant over time, with periods of stagnation of approximately 5 years. The authors appear grouped in relatively independent clusters, thus suggesting a low level of collaboration between those devoted to the Sigma-1 receptor. Furthermore, as evidenced by the number of recent articles, only one pharmaceutical company, developing selective Sigma-1 receptor drug ligands, is currently actively publishing in the field. Additionally, the very nature of the Sigma-1 receptor may also be influencing the low success in transforming basic research into new drugs with a clear therapeutic potential. It is common in papers to describe the Sigma-1 receptor as an “enigmatic protein whose molecular mechanism of action remains elusive.” The most cited article we found in this bibliometric analysis (Hayashi and Su, 2007) proposed that the Sigma-1 receptor acts as a molecular chaperone and therefore is not a traditional receptor. Chaperones ensure that all proteins obtain their correct folding and functionalities in the right localization at the right time. They recognize and bind their protein clients in conformational ensembles that are locally highly dynamic and interconvert, while in other cases clients bind in unique conformations (Hiller, 2019). Thus, to transform basic research into new drugs with a clear therapeutic potential, the intrinsic difficulty when trying to understand the mysteries of this unique ligand-regulated molecular chaperone should be considered in drug discovery programs.

In summary, a greater interest and involvement of the scientific community for this enigmatic chaperone, accompanied by a parallel increase in the scientific production would help, hopefully in coming years, to the discovery of new functions and deepening in those already known. Additional boost needed to improve research performance are likely to come from new conceptual frameworks and breakthrough discoveries derived from recent and future advances in the “chaperone field,” and from collaborative, synergistic initiatives by combining resources and knowhow from different laboratories to overcome the limitations of the individual approaches. This study may provide a valuable basis for identifying important topics for future research, and create opportunities for collaboration between research groups with complementary scientific interest in the field of Sigma-1 receptor.

## Data Availability

The datasets generated for this study are available on request to the corresponding author.

## Author Contributions

EP-S and LR designed the study, performed the search, analyzed the data, and wrote the paper.

### Conflict of Interest Statement

LR and EP-S are employees of ESTEVE Pharmaceuticals.
